# Supplementary material for: The EstroGene2.0 database for endocrine therapy response and resistance in breast cancer
Source: NPJ Breast Cancer. 2024 Dec 19;10:106. doi: 10.1038/s41523-024-00709-4 (PMC11659402; doi:10.1038/s41523-024-00709-4)
Supplement: Supplementary file 1 — Supplementary Information [file 41523_2024_709_MOESM1_ESM.pdf]

The EstroGene2.0 database for endocrine therapy response and resistance in breast cancer

Supplementary Figures

Supplementary Figure 1

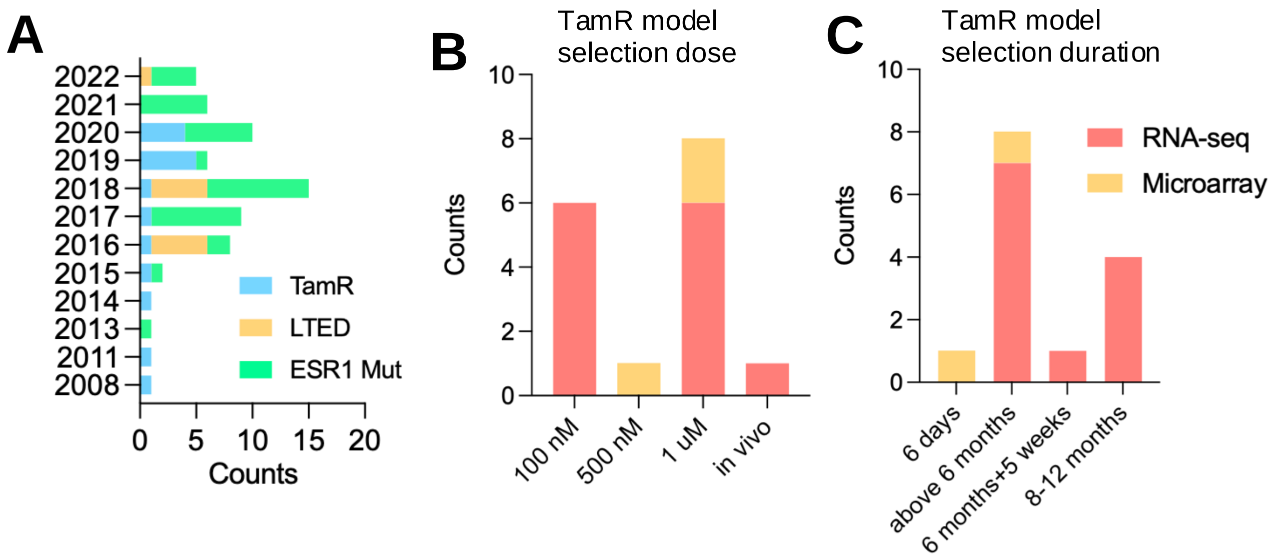

Supplementary Figure 1 (related to Fig. 1)
A. Stacked histogram showing the metadata separated by technologies and three endocrine resistant model types.
B and C. Stacked histogram summarizing the dose (B) and duration (C) of tamoxifen resistant model selection separated by technologies.

Supplementary Figure 2

A

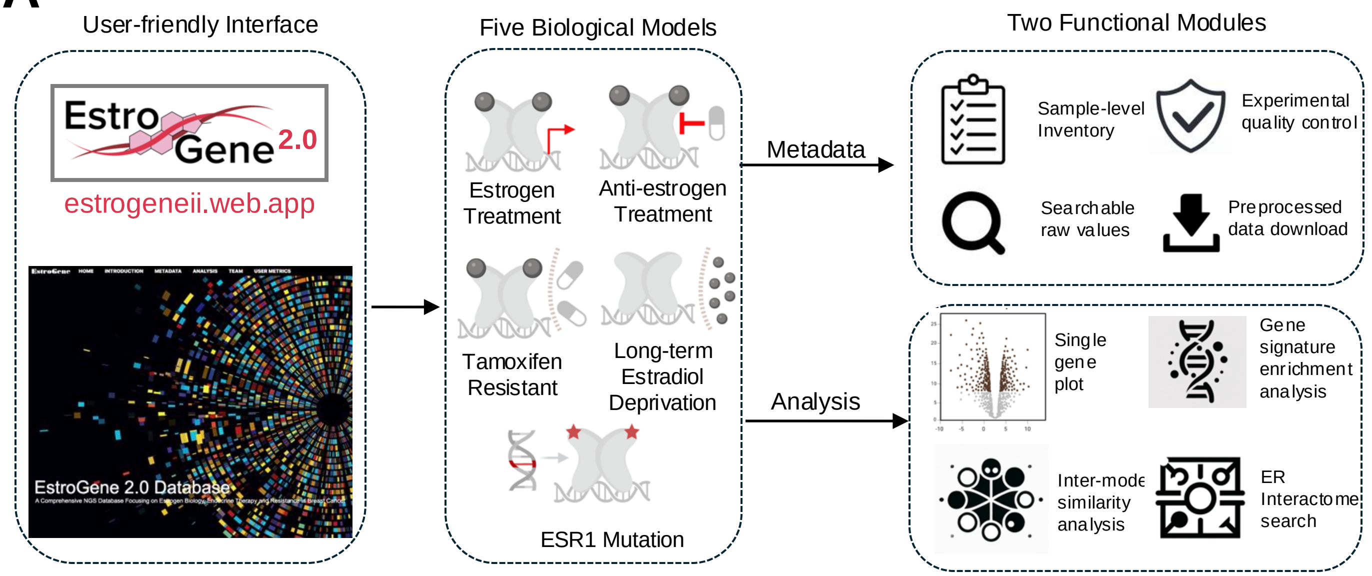

B

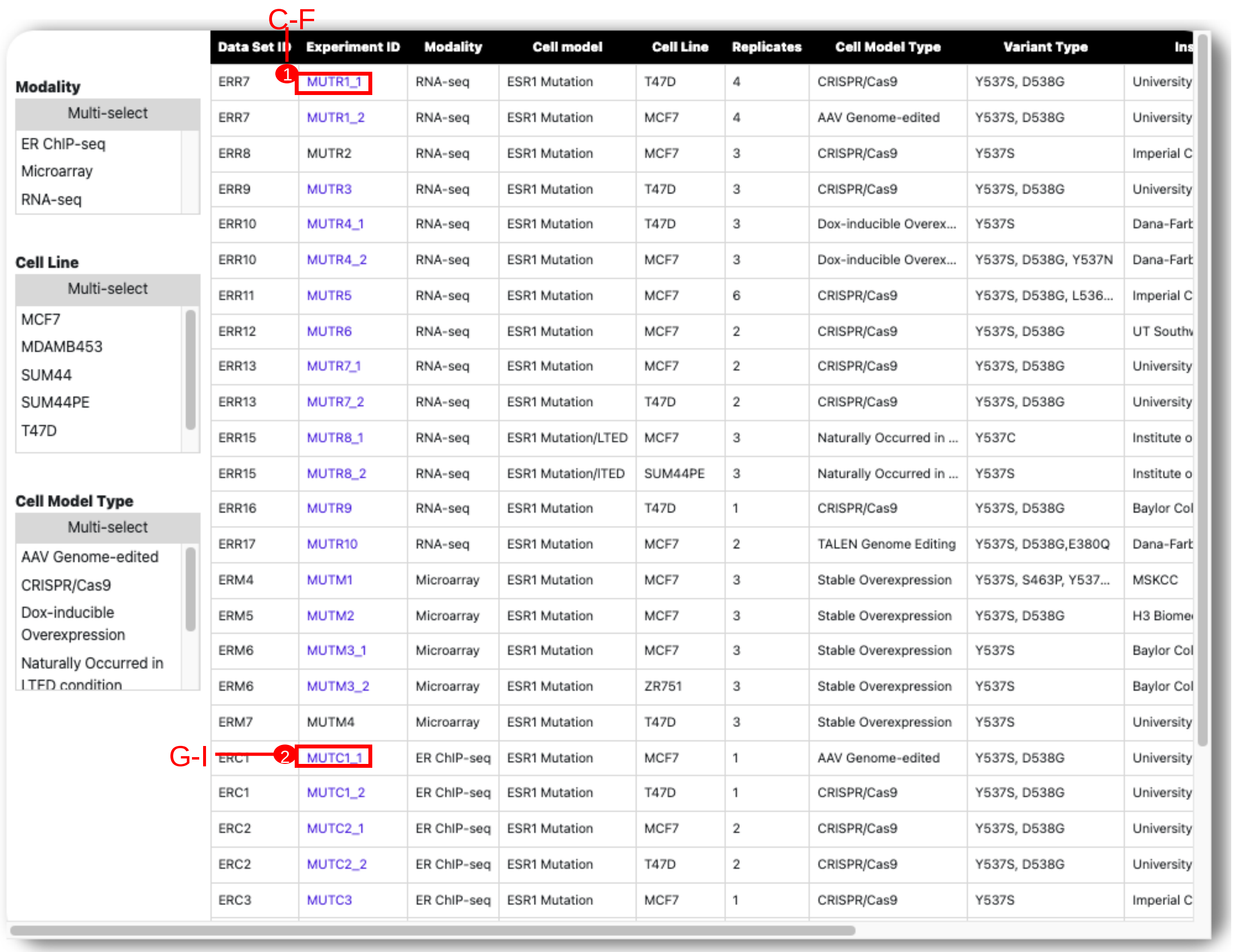

Data Set ID	Experiment ID	Modality	Cell model	Cell Line	Replicates	Cell Model Type	Variant Type	Ins
ERR7	MUTR1_1	RNA-seq	ESR1 Mutation	T47D	4	CRISPR/Cas9	Y537S, D538G	University
ERR7	MUTR1_2	RNA-seq	ESR1 Mutation	MCF7	4	AAV Genome-edited	Y537S, D538G	University
ERR8	MUTR2	RNA-seq	ESR1 Mutation	MCF7	3	CRISPR/Cas9	Y537S	Imperial C
ERR9	MUTR3	RNA-seq	ESR1 Mutation	T47D	3	CRISPR/Cas9	Y537S, D538G	University
ERR10	MUTR4_1	RNA-seq	ESR1 Mutation	T47D	3	Dox-inducible Overex...	Y537S	Dana-Farb
ERR10	MUTR4_2	RNA-seq	ESR1 Mutation	MCF7	3	Dox-inducible Overex...	Y537S, D538G, Y537N	Dana-Farb
ERR11	MUTR5	RNA-seq	ESR1 Mutation	MCF7	6	CRISPR/Cas9	Y537S, D538G, L536...	Imperial C
ERR12	MUTR6	RNA-seq	ESR1 Mutation	MCF7	2	CRISPR/Cas9	Y537S, D538G	UT Southw
ERR13	MUTR7_1	RNA-seq	ESR1 Mutation	MCF7	2	CRISPR/Cas9	Y537S, D538G	University
ERR13	MUTR7_2	RNA-seq	ESR1 Mutation	T47D	2	CRISPR/Cas9	Y537S, D538G	University
ERR15	MUTR8_1	RNA-seq	ESR1 Mutation/LTED	MCF7	3	Naturally Occurred in ...	Y537C	Institute o
ERR15	MUTR8_2	RNA-seq	ESR1 Mutation/ITED	SUM44PE	3	Naturally Occurred in ...	Y537S	Institute o
ERR16	MUTR9	RNA-seq	ESR1 Mutation	T47D	1	CRISPR/Cas9	Y537S, D538G	Baylor Col
ERR17	MUTR10	RNA-seq	ESR1 Mutation	MCF7	2	TALEN Genome Editing	Y537S, D538G,E380Q	Dana-Farb
ERM4	MUTM1	Microarray	ESR1 Mutation	MCF7	3	Stable Overexpression	Y537S, S463P, Y537...	MSKCC
ERM5	MUTM2	Microarray	ESR1 Mutation	MCF7	3	Stable Overexpression	Y537S, D538G	H3 Biome
ERM6	MUTM3_1	Microarray	ESR1 Mutation	MCF7	3	Stable Overexpression	Y537S	Baylor Col
ERM6	MUTM3_2	Microarray	ESR1 Mutation	ZR751	3	Stable Overexpression	Y537S	Baylor Col
ERM7	MUTM4	Microarray	ESR1 Mutation	T47D	3	Stable Overexpression	Y537S	University
ERC1	MUTC1_1	ER ChIP-seq	ESR1 Mutation	MCF7	1	AAV Genome-edited	Y537S, D538G	University
ERC1	MUTC1_2	ER ChIP-seq	ESR1 Mutation	T47D	1	CRISPR/Cas9	Y537S, D538G	University
ERC2	MUTC2_1	ER ChIP-seq	ESR1 Mutation	MCF7	2	CRISPR/Cas9	Y537S, D538G	University
ERC2	MUTC2_2	ER ChIP-seq	ESR1 Mutation	T47D	2	CRISPR/Cas9	Y537S, D538G	University
ERC3	MUTC3	ER ChIP-seq	ESR1 Mutation	MCF7	1	CRISPR/Cas9	Y537S	Imperial C

Supplementary Figure 2 (Continued)

C

GSM ID	Cell Line	Sample ID	ESR1 Genotype	GREB1
GSM2392590	T47D	MUTR1_1_DG1	D538G	6.710
GSM2392591	T47D	MUTR1_1_DG2	D538G	6.505
GSM2392592	T47D	MUTR1_1_DG3	D538G	6.628
GSM2392593	T47D	MUTR1_1_DG4	D538G	6.365
GSM2392582	T47D	MUTR1_1_WT1	WT	5.806
GSM2392583	T47D	MUTR1_1_WT2	WT	5.689
GSM2392584	T47D	MUTR1_1_WT3	WT	5.776
GSM2392585	T47D	MUTR1_1_WT4	WT	5.770
GSM2392586	T47D	MUTR1_1_YS1	Y537S	7.385
GSM2392587	T47D	MUTR1_1_YS2	Y537S	7.332
GSM2392588	T47D	MUTR1_1_YS3	Y537S	7.697
GSM2392589	T47D	MUTR1_1_YS4	Y537S	7.420

D

Comparison	log2 Fold Change	Adjusted P-value
D538G over WT	0.8002	3.18e-41
Y537S over WT	1.713	5.43e-79

E

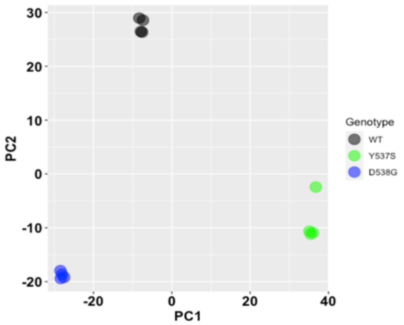

F

- MUTR1_1_D538G_over_WT_DEseq_output.csv
- MUTR1_1_log2CPM_Expression_Matrix.csv
- MUTR1_1_Y537S_over_WT_DEseq_output.csv

G

SRR ID	Cell Line	Sample ID	ESR1 Genotype	Mapped Reads(M)	Total Peaks
SRR8444288	MCF7	MCF7_D538G_Vehicle	D538G	17.36	399
SRR8444282	MCF7	MCF7_WT_Vehicle	WT	19.59	26
SRR8444285	MCF7	MCF7_Y537S_Vehicle	Y537S	18.18	265

H

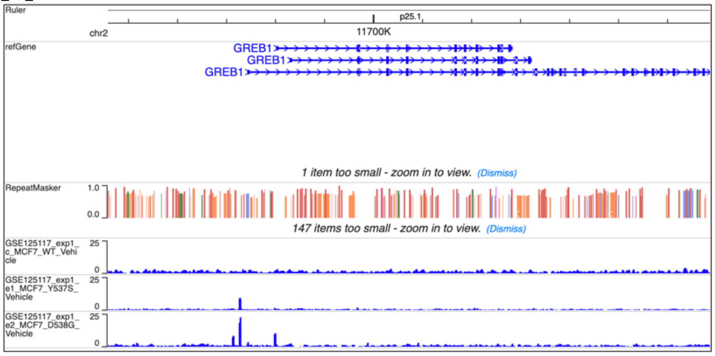

I

- MUTC1_1_MCF7_D538G_Vehicle_peaks.narrowPeak
- MUTC1_1_MCF7_WT_Vehicle_peaks.narrowPeak
- MUTC1_1_MCF7_Y537S_Vehicle_peaks.narrowPeak
- MUTC1_1_read.counts.rpkm.csv

Supplementary Figure 2.
A. Schematic overview of EstroGene2.0 website construction.
B. A screen shot from EstroGene2.0 browser of “METADATA” tab of ESR1 mutant models. Red dots indicate the two example data sets elaborated in the following panels.
C-F. Screen shot from EstroGene2.0 browser of searchable “Gene Expression Matrix” panel (C),”Differentially Gene Expression” panel (D), ”Principal Component Analysis” (E) and “Download” tabs for MUTR1_1 RNA-seq experiment (F), as an example of gene expression profiling.
H-I. Screen shot from EstroGene2.0 browser of “Experimental Metadata” (G), “Genomic Track Veiw” (H) and “Download” tabs for MUTC1_1 ER ChIP-seq experiment (I), as an example of ChIP-seq profiling.

Supplementary Figure 3

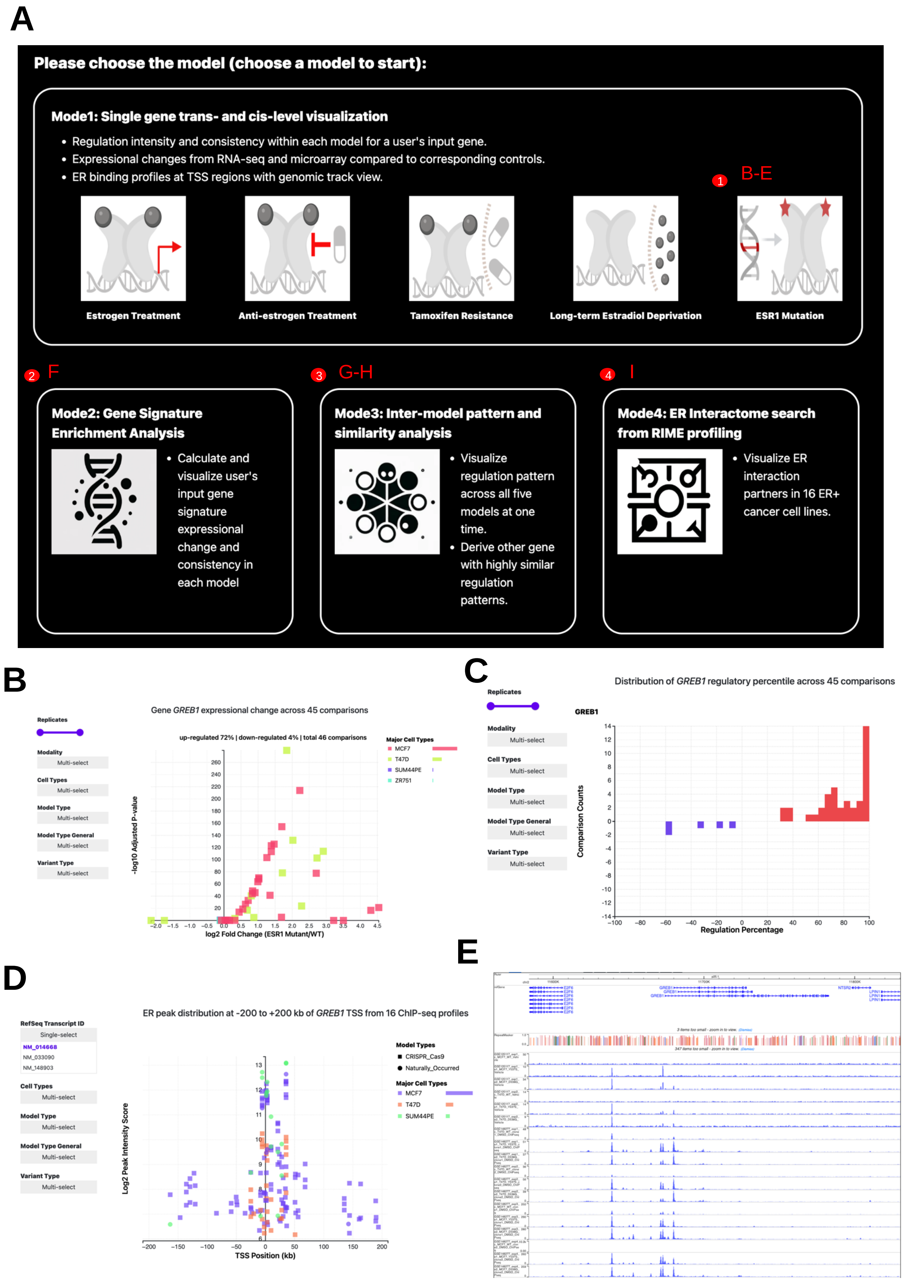

Supplementary Figure 3 (Continued)

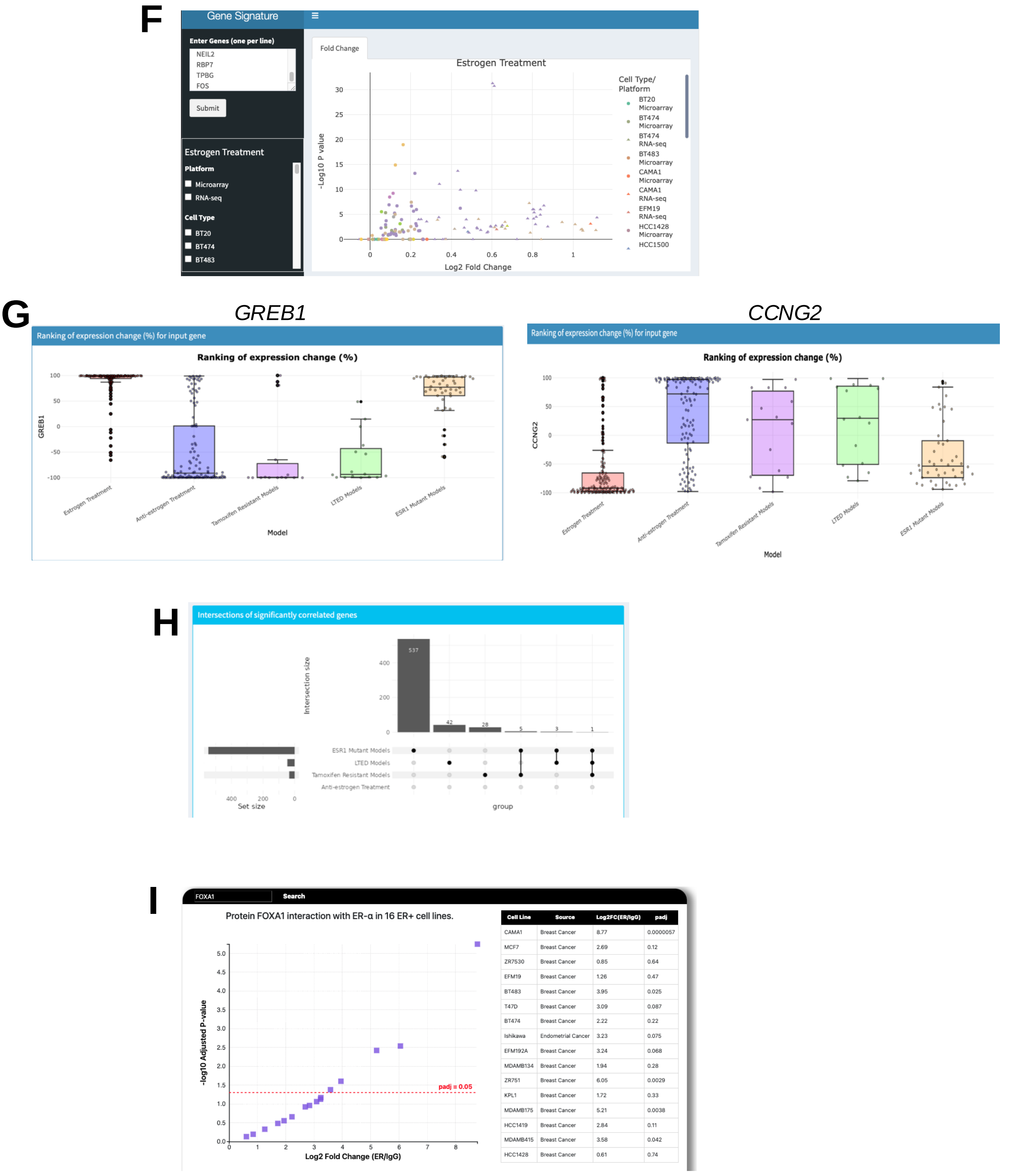

Supplementary Figure 3.
A. A screen shot from EstroGene2.0 browser of “ANALYSIS” tab. Red dots indicate the sections elaborated in the following panels.
B-E. Screen shots from EstroGene2.0 browser of “Volcano Plot” (B) and “Percentile Plot” (C) from gene entry “GREB1” in ESR1 mutation section, and ER ChIP-seq data visualization of “TSS Region View” (D) and “Genomic Track View” (E) from from gene entry “GREB1” in ESR1 mutation section.
F. A screen shot of output from EstroGene2.0 browser of Mode2: Gene Signature Enrichment Analysis, using EstroGene meta-signature as an input and the enrichment score in estrogen treatment experiments were plotted.
G and H. Screen shots of output from EstroGene2.0 browser of Mode3: Inter-model pattern and similarity analysis, using gene GREB1 as input. Output indicates the box plot view of overall regulatory percentile of each comparisons across five different sections for *GREB1* and *CCNG2* (G) and significantly similar genes shared across all four sections (H) for *GREB1*.
I. A screen shot of output from EstroGene2.0 browser of Mode4: ER Interactome search from RIME profiling, using FOXA1 as a default input. Volcano plot depicting the log2FC and -log10padj normalized to IgG control in 16 ER+ cancer cell lines.

Supplementary Figure 4

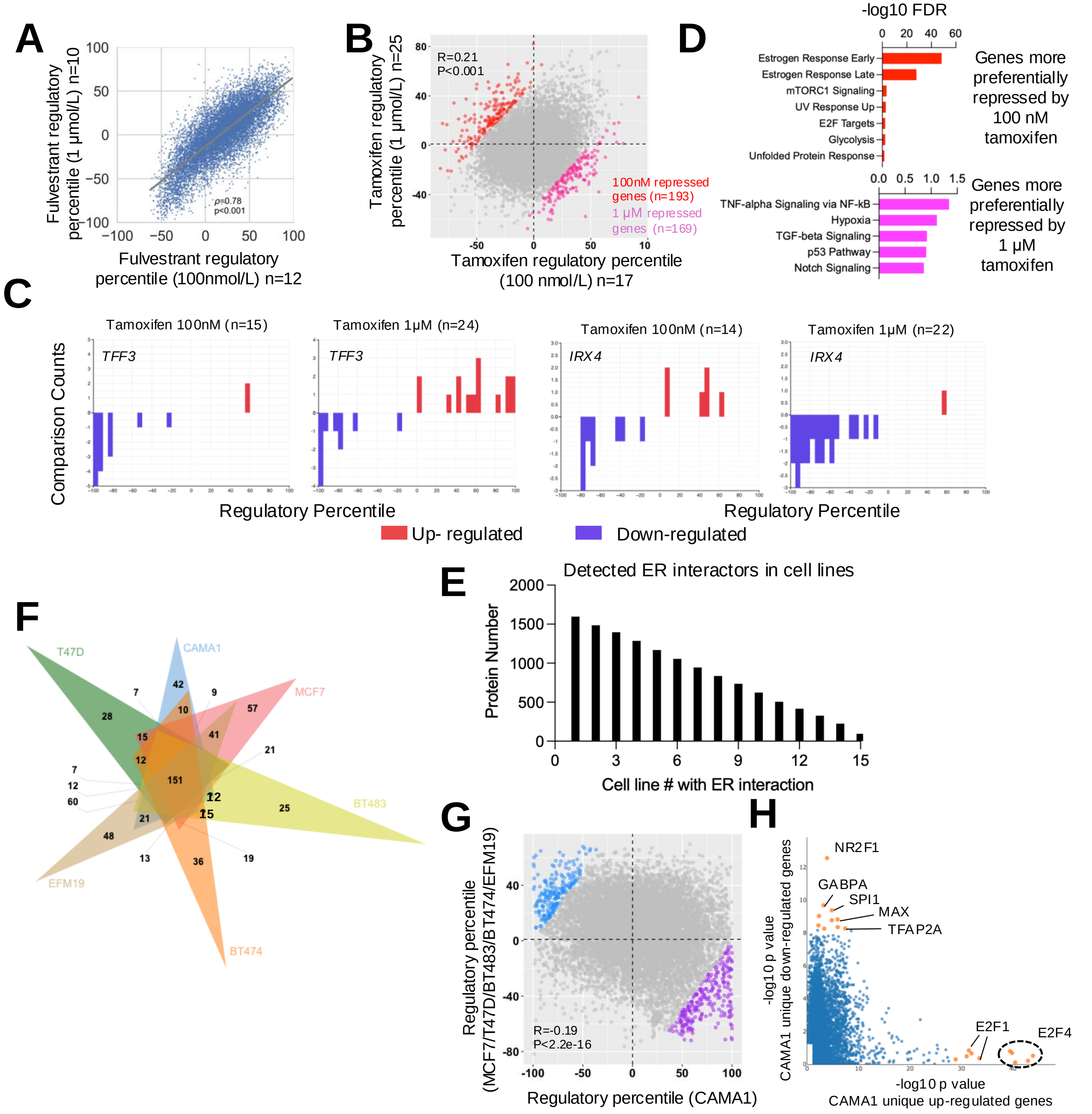

Supplementary Figure 4 (related to Fig. 2)

A and B. Scattered plots representing the Pearson correlation of regulatory percentile between 100 nM and 1 μM fulvestrant (A) and tamoxifen (B) treatment. For tamoxifen treatment experiments, genes that more pronouncedly repressed by high and low dose were highlighted in pink and red respectively. (|delta regulatory percentile| >100 between the two models).

C. Bar plot showing the distribution of regulatory percentile of TFF3 and IRX4 in all the comparisons from 100nM and 1 μM tamoxifen treatment. Percentiles are ranged between -100 to +100 and larger number indicates stronger regulation.

D. Bar graph showing the significantly enriched Hallmark pathways in high and low tamoxifen-preferentially repressed genes indicated in B.

E. Bar plot representing the detected ER interactor numbers by RIME (log2FC>5, padj<0.05 to IgG) in number of breast cancer cell lines cumulative from 1-15.

F. Venn diagram showing the overlapping of ER interactors from RIME experiment (log2FC>5, padj<0.05 to IgG) among CAMA1, MCF7, T47D, BT483, BT474 and EFM19 cells.

G. Scattered plots representing the Pearson correlation of regulatory percentile between CAMA1 and average of MCF7, T47D, BT483, BT474 and EFM19 cells under tamoxifen treatment. Genes that are differentially regulated by CAMA1 and other five cell lines were highlighted in blue and purple respectively. (|delta regulatory percentile| >100 between the two subgorups).

H. Scattered plot showing the correlation of -log10 p values of LISA predicted regulators from differentially regulated genes between CAMA1 and MCF7, T47D, BT483, BT474 and EFM19 cells in F. Only significantly enriched regulators were shown and top targets skewed to each side were labelled.

Supplementary Figure 5

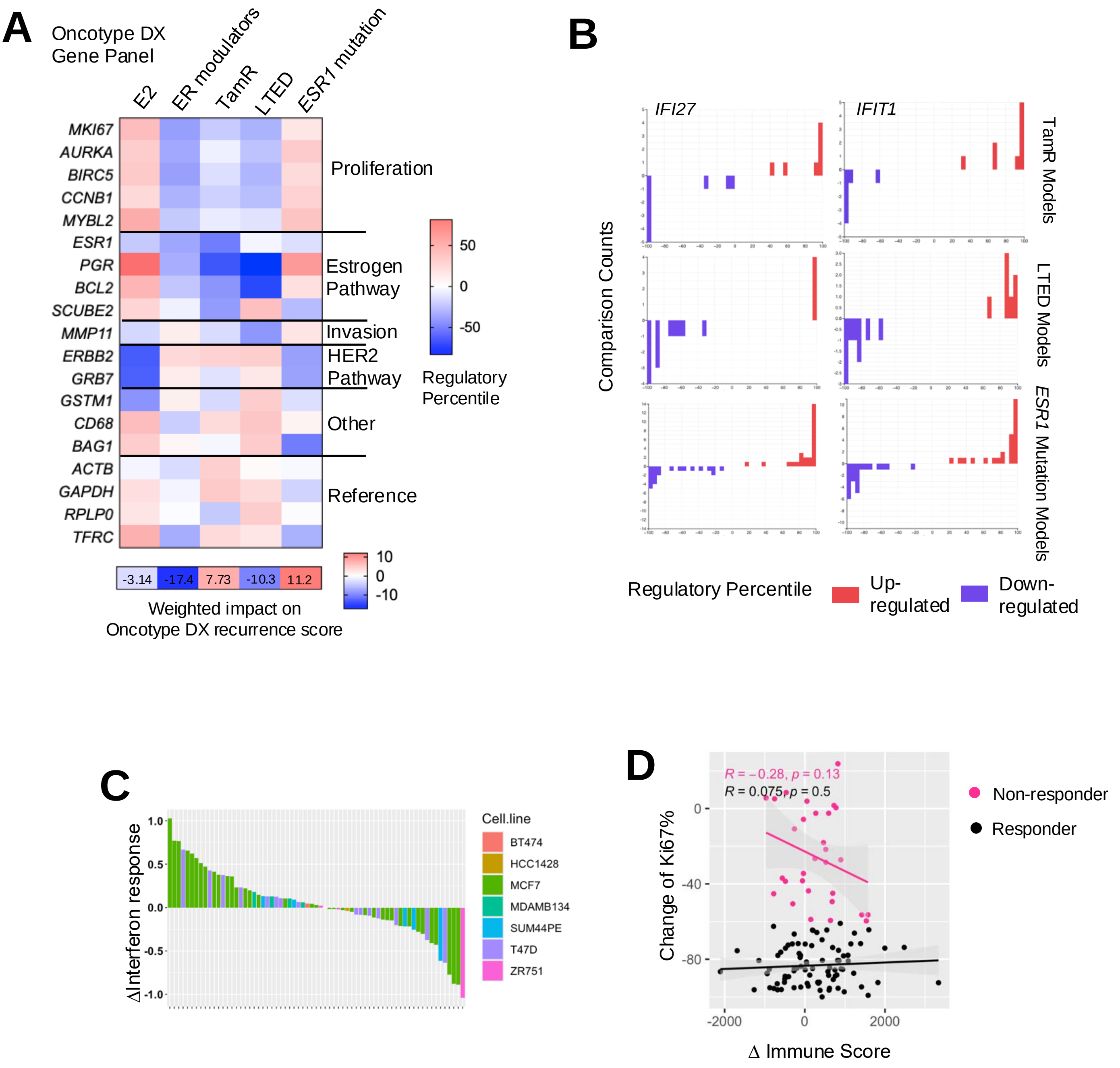

Supplementary Figure 5 (related to Fig. 3)

A. Heatmap illustrating the average regulatory percentile changes of Oncotype DX gene panel under the five indicated conditions normalized to each corresponding controls. Overall impact on Oncotype DX recurrence scores were derived by considering the weighted value based on the original formula.

B. Bar plot showing the distribution of regulatory percentile of IFI27 and IFIT1 in all the comparisons from TamR, LTED and ESR1 mutations respectively. Percentiles are ranged between -100 to +100 and larger number indicates stronger regulation.

C. Bar graph showing the the alteration of GSVA enrichment scores of mean of interferon response α and γ signatures in all the comparisons from endocrine resistant models. Color code indicate specific cell lines used.

D. Scatter plots representing the Pearson correlation between treatment-caused changes of immune infiltration scores towards treatment-induced changes of KI67% of each patient separated by responders and non-responders.

Supplementary Figure 6

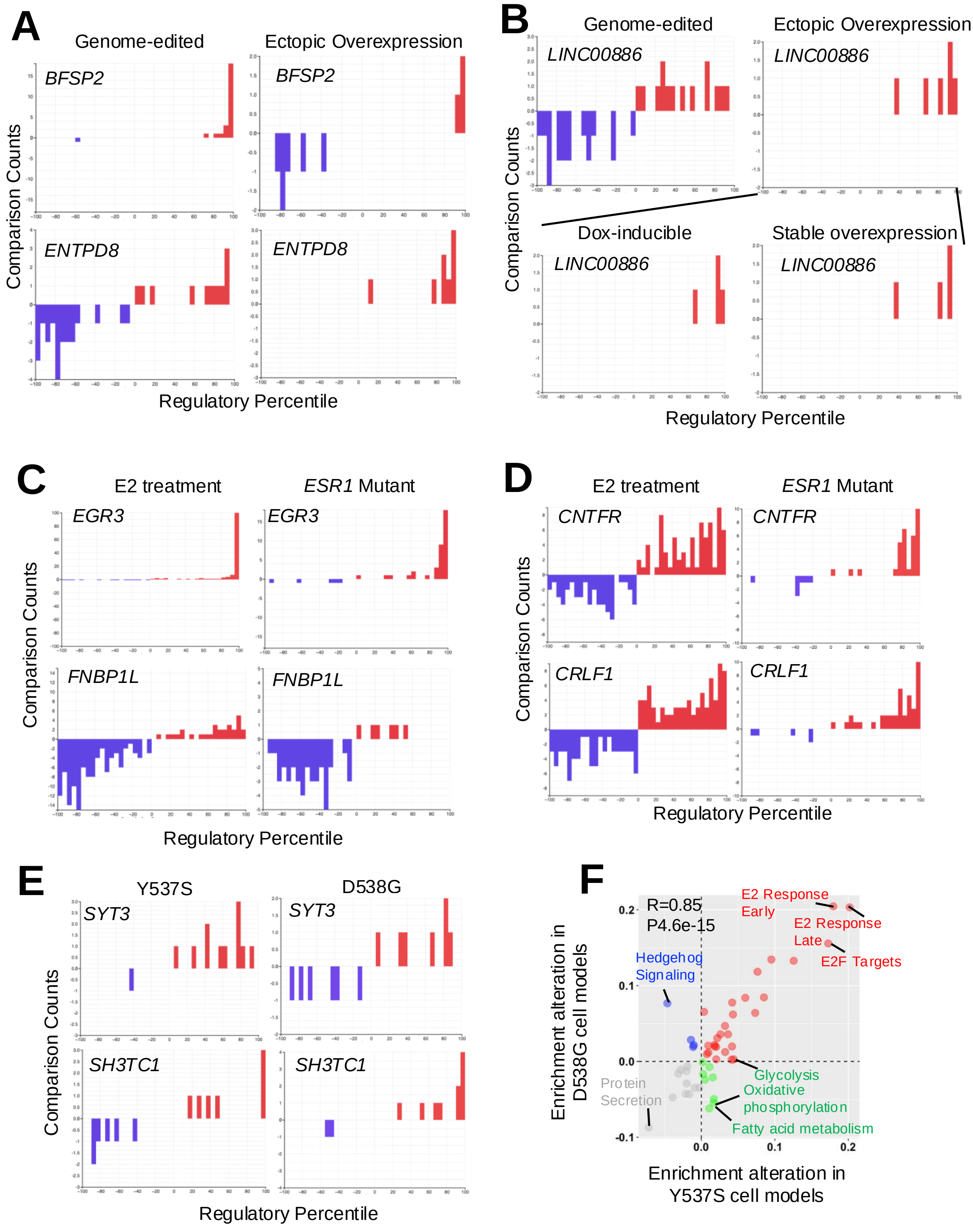

Supplementary Figure 6 (related to Fig. 4)

A-E. Bar plot showing the distribution of regulatory percentile of indicated genes in the selected comparisons from endocrine resistant cells. Percentiles are ranged between -100 to +100 and larger number indicates stronger regulation.

F. Scattered plot showing Pearson correlation between Hallmark signature enrichment score alteration in Y537S and D538G models normalized to the corresponding WT controls.

Supplementary Figure 7

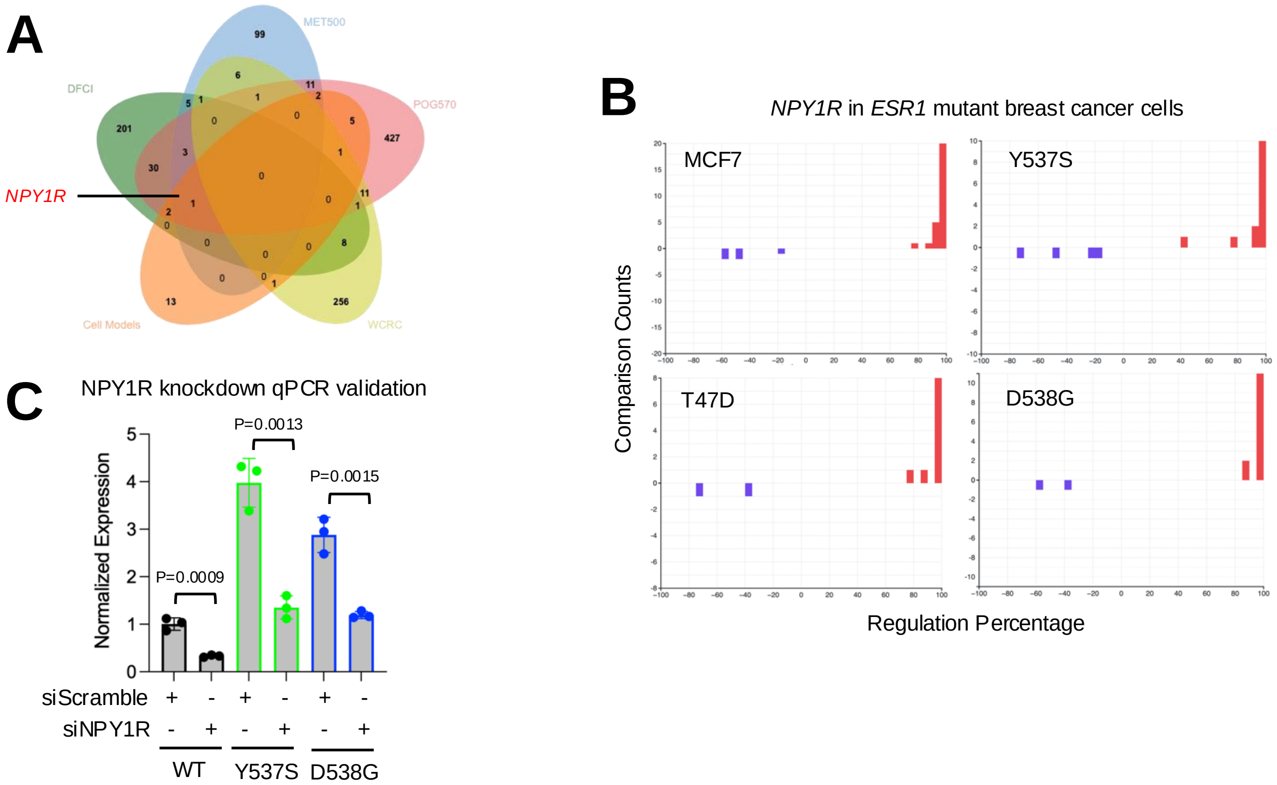

Supplementary Figure 7 (related to Fig. 5)

A. Venn diagram showing the overlap of top consistent upregulated genes in ESR1 mutant cell models cell models (from Fig. 7A) and significantly upregulated genes in ESR1 mutant tumors from four ER+ metastatic cohorts.

B. Bar plot showing the distribution of regulatory percentile of NPY1R in the selected comparisons from ESR1 mutation cell models. Percentiles are ranged between -100 to +100 and larger number indicates stronger regulation.

C. Box plots showing the relative expression of NPY1R in genome-edited MCF7 and T47D ESR1 mutant cells and WT cells in the presence of scramble and NPY1R siRNA transfection . ∆∆Ct method was used and p values were calculated using one-way ANOVA.

Supplementary Data Legends

Supplementary Data 1. Inventory of dataset documentation of EstroGene2.0 database.

Each spreadsheet represents a detailed inventory of a catalog of EstroGene2.0 including dataset ID, technologies, cell line, replicates, treatment or engineering details as well as the original publication and GEO deposition.

Supplementary Data 2. Gene lists used in this study.

Each column represents a list of gene sets that were computed from this study mentioned in the manuscript.

Supplementary Data 3. Details of ER RIME profiling.

Spreadsheet 1 shows the raw log2 fold change and adjusted p values of each protein between ER and IgG control in all 16 cell lines. Spreadsheet 2 shows the binary determination of whether a protein interacts with ER in each cell line. Interaction was defined as log2FC>5 and padj<0.05 compared to IgG. “1” and “0” refers to positive and negative for ER interaction detection.

Supplementary Data 4. CAMA1 specific regulator and ER interactor list.

Each column represents the unique ER interactors and predicted regulators for CAMA1 unique up and down-regulated genes detailed in Figure 2L.
